# Potential of biomarkers during pharmacological therapy setting for postmenopausal osteoporosis: a systematic review

**DOI:** 10.1186/s13018-021-02497-0

**Published:** 2021-05-31

**Authors:** Filippo Migliorini, Nicola Maffulli, Filippo Spiezia, Giuseppe Maria Peretti, Markus Tingart, Riccardo Giorgino

**Affiliations:** 1 Department of Orthopaedic Surgery, University Clinic Aachen, RWTH Aachen University Clinic, Pauwelsstraße 30, 52074 Aachen, Germany; 2 Department of Medicine, Surgery and Dentistry, University of Salerno, Via S. Allende, 84081 Baronissi, SA Italy; 3 School of Pharmacy and Bioengineering, Keele University Faculty of Medicine, Thornburrow Drive, Stoke on Trent, Newcastle-under-Lyme, England; 4 Queen Mary University of London, Barts and the London School of Medicine and Dentistry, Centre for Sports and Exercise Medicine, Mile End Hospital, 275 Bancroft Road, London, E1 4DG England; 5 Department of Orthopedics and Trauma Surgery, Ospedale San Carlo di Potenza, Potenza, Italy; 6 Department of Biomedical Sciences for Health, University of Milan, Milan, Italy; 7 Department of Orthopaedic Surgery, IRCCS Istituto Ortopedico Galeazzi, Milan, Italy

**Keywords:** Osteoporosis, Biomarkers, bALP, PINP, bCTx, NTx

## Abstract

**Background:**

Biochemical markers of bone turnover (BTMs), such as the bone alkaline phosphatase (bALP), procollagen type I N propeptide (PINP), serum cross-linked C-telopeptides of type I collagen (bCTx), and urinary cross-linked N-telopeptides of type I collagen (NTx), are used to manage therapy monitoring in osteoporotic patients. This systematic review analyzed the potential of these BMTs in predicting the clinical outcomes in terms of BMD, t-score, rate of fractures, and adverse events during the therapy setting in postmenopausal osteoporosis.

**Methods:**

All randomized clinical trials (RCTs) reporting data on biomarkers for postmenopausal osteoporosis were accessed. Only articles reporting quantitative data on the level of biomarkers at baseline and on the outcomes of interest at the last follow-up were eligible.

**Results:**

A total of 36,706 patients were retrieved. Greater values of bALP were associated with a greater rate of vertebral (P = 0.001) and non-vertebral fractures (P = 0.0001). Greater values of NTx at baseline were associated with a greater rate of adverse events at the last follow-up (P = 0.02). Greater values of CTx at baseline were associated with a greater rate of adverse events leading to discontinuation (P = 0.04), gastrointestinal adverse events (P = 0.0001), musculoskeletal adverse events (P = 0.04), and mortality (P = 0.04). Greater values of PINP at baseline were associated with greater rates of gastrointestinal adverse events (P = 0.02) at the last follow-up.

**Conclusion:**

The present analysis supports the adoption of BMTs during pharmacological therapy setting of patients suffering from osteoporosis.

**Level of evidence:**

I, systematic review of RCTs

## Introduction

The management of osteoporosis represents an important therapeutic challenge for the global health system and constitutes a considerable health expenditure [1–3]. In addition, increasing in average age [4, 5] could have a significant impact on healthcare costs for the wide range of drugs that are used to manage osteoporotic patients [6–8]. Different drugs and administration methods have been shown to be more effective than others in the prevention of a certain complication or clinical outcomes such as BMD, t-score, rate of fractures, and adverse events [9–14]. However, prevention of complication along the natural history of the disease is not an easy task to obtain [15, 16].

Biochemical markers of bone turnover (BTMs) have gained popularity for their ability to provide specific and dynamic indications of bone turnover mechanisms in the delicate balance between formation and resorption [17–19]. More precisely, serum bone alkaline phosphatase (bALP) and procollagen type I N propeptide (PINP) are considered biomarkers of bone ossification, while serum cross-linked C-telopeptides of type I collagen (bCTx) and urinary cross-linked N-telopeptides of type I collagen (NTx) are considered indicators of bone resorption [17, 20, 21]. For their role in bone turnover, these BMTs could be used as a tool for monitoring therapy in osteoporosis [22–24]. With these assumptions, a systematic review has been performed to identify in these markers a predictor role for complications in the osteoporotic patient, and their ability to intervene with the most effective drug for the individual patient.

The purpose of the present study was to establish the potential of bALP, PINP, bCTx, and NTx in predicting the clinical outcomes in terms of BMD, t-score, rate of fractures, and adverse events during the therapy setting in patients with postmenopausal osteoporosis.

## Material and methods

### Search strategy

The present study followed the Preferred Reporting Items for Systematic Reviews and Meta-Analyses (PRISMA) [25]. The PICOT framework was structured as follows:

- P (problem): postmenopausal osteoporosis
- I (intervention): bALP, PINP, bCTx, and NTx
- C (control): therapy setting
- O (outcomes): BMI, fractures, adverse events
- T (timing): ≥ 6 months of follow-up

Two authors (FM;RG) independently performed the literature search. In December 2020, the following databases were accessed: PubMed, Google Scholar, Embase, and Scopus. No time constraints were set for the database search. The following keywords were used in combination: *osteoporosis, treatment, management, drug, pharmacology, pharmacological, medicament, mineral, density, bone, BMD, bone alkaline phosphatase, ALP, procollagen type I N propeptide, PINP, serum cross-linked C-telopeptides of type I collagen, CTx, urinary cross-linked N-telopeptides of type I collagen, NTx, premenopausal, spine, pathological, fragility, fractures, hip, vertebral, disability, adverse events, Bisphosphonates, Denosumab, Romosozumab, Clodronate, Raloxifene, Teriparatide, Alendronate, Risedronate, Zoledronate, Ibandronate, Etidronate, PTH, osteoblast, osteoclast.* The resulting articles were screened by the same authors. The full text of the articles of interest was accessed. A cross-reference of the bibliographies was also performed.

### Eligibility criteria

All randomized clinical trials (RCTs) reporting data on biomarkers for postmenopausal osteoporosis were accessed. According to the authors’ language capabilities, articles in English, French, German, Italian, Portuguese, and Spanish were eligible. Only studies of level I evidence, according to the Oxford Centre of Evidence-Based Medicine (OCEBM) [26] were considered. Articles reporting data on patients with secondary osteoporosis were excluded. Studies concerning patients with tumors and/or bone metastases were also not included. Studies reporting data on patients with iatrogenic-induced menopausal were not included, nor those on pediatric and/or adolescent patients. Studies regarding selected patients undergoing immunosuppressive therapies or organ transplantation were not considered. Studies reporting data on combined therapies with multiple drugs were not eligible. Studies with follow-up shorter than 6 months were not eligible, nor were those involving less than 10 patients. Studies reporting data of combined therapy with multiple anti-osteoporotic drugs were also not included. Only articles reporting quantitative data on the level of biomarkers at baseline and on the outcomes of interest were eligible. Missing data under these endpoints warranted the exclusion from the present work.

### Data extraction and outcomes of interests

Two authors (FM;RG) performed data extraction. Study generalities (author, year, journal, duration of the follow-up, daily calcium and vitamin D supplementation, treatment) and patient baseline demographic information were collected: number of samples, mean age, mean bone mass index (BMI), mean BMD (overall, spine, hip, femur neck), t score (spine, hip, femur), and number of previous vertebral and non-vertebral fragility fractures. Data concerning the following endpoints were collected at the last follow-up: mean BMD (overall, spine, hip, femur neck), rate of vertebral, non-vertebral, femoral, hip fragility fractures, and body height. Data concerning the following adverse events at the last follow-up were collected: overall adverse events, serious adverse events and those leading to study discontinuation, gastrointestinal events, musculoskeletal events, rate of osteonecrosis, and mortality. Data concerning bALP, PINP, bCTx, and NTx were extracted at baseline and last follow-up. The outcomes of interest were to assess the association between biomarkers and patient characteristics, bone mass density, and adverse events at the last follow-up.

### Methodological quality assessment

The methodological quality assessment was made through the risk of bias graph tool of the Review Manager Software (The Nordic Cochrane Collaboration, Copenhagen). The following risks of bias were evaluated: selection, detection, performance, reporting, attrition, and other sources of bias.

### Statistical analysis

The statistical analyses were performed by the main author (FM). The IBM SPSS software version 25 was used to assess data at baseline. Data distribution was evaluated using the Shapiro–Wilk test. Normally distributed data were evaluated using mean and standard deviation (SD), while median and interquartile range (IQR) were calculated for non-parametric data. The Student *T*-test was used to assess significance for parametric data, while the Mann–Whitney *U*-test for non-parametric variables. Values of P < 0.05 are considered statistically significant. Multiple linear pairwise correlations  were performed to assess associations between the value of the biomarkers at baseline and patient demographics, bone mass density, and adverse events at the last follow-up. The STATA Software/MP version 16 (StataCorporation, College Station, TX, USA) is used for the statistical analyses. A multiple linear model regression analysis through the Pearson product–moment correlation coefficient (*r*) was used. The Cauchy–Schwarz formula was used for inequality: +1 is considered as positive linear correlation, while −1 a negative one. Values of 0.1< | *r* | < 0.3, 0.3< | *r* | < 0.5, and | *r* | > 0.5 were considered to have weak, moderate, and strong correlation, respectively. The overall significance was assessed through the χ^2^ test, with values of P < 0.05 considered statistically significant.

## Results

### Search result

**Fig. 1:**
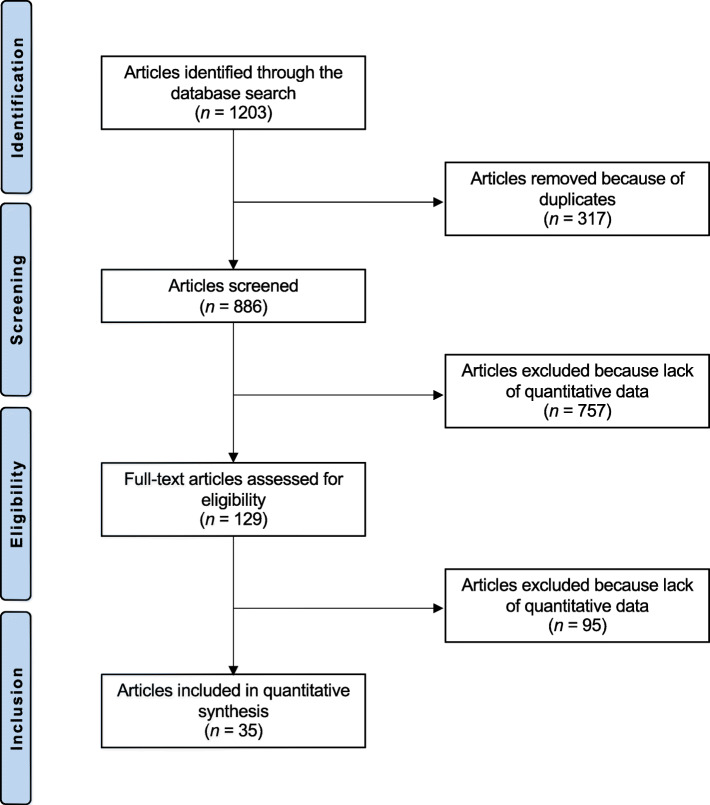
Flow chart of the literature search

The literature search resulted in 1203 studies. Of them, 317 were duplicates. A further 757 articles were excluded because of study design (N = 221), non-clinical studies (N = 319), secondary osteoporosis (N = 87), small population or short follow-up (N = 15), multiple therapies (N = 33), language limitations (N = 5), uncertain results (N = 11), and others (N = 66). Another 95 articles were excluded because of data under the outcomes of interest missing. Finally, 35 RCTs were eligible for the present study (Fig. 1).

### Methodological quality assessment

**Fig. 2:**
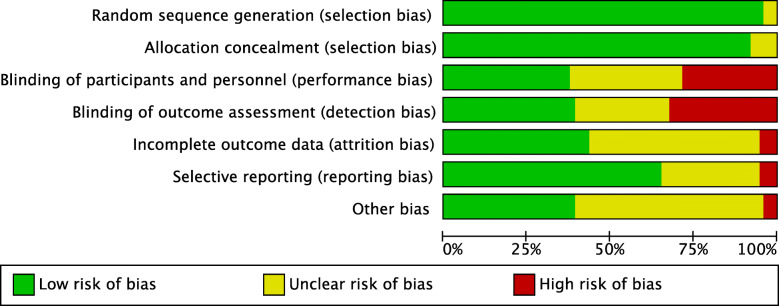
Methodological quality assessment

Given the exclusive inclusion of only RCTs, the risk of selection bias was low. Most of the studies were single and double blinded, leading to moderate-low risk of detection and performance biases. Overall, the high quality of the studies leads to a low risk of attrition and reporting bias. Concluding, the results of the review evaluation about each risk of bias item for each individual included study (Fig. 2) were low to moderate, leading to a good assessment of the methodology.

### Patient demographics

A total of 36,706 patients were included. The median age was 67 (IQR 5), the median BMI 25.4 (IQR 1.9). The median vertebral BMD was 0.84 (IQR 0.17), hip BMD 0.74 (IQR 0.11), and femur BMD 0.64 (IQR 0.03). The ANOVA test found optimal within-group variance concerning age, BMI, and BMDs (P > 0.1). Generalities and patient baseline data of the included studies are shown in detail in Table 1.

**Table 1:** Generalities and patient baseline data of the included studies

Author, year	Journal	Mean follow-up (months)	Mean calcium daily supplement (mg)	Mean vit D daily supplement (UI)	Treatment	Administration	Samples (***n***)	Mean age	Mean BMI (kg/m^**2**^)	Mean BMD spine (g/cm^**2**^)	Mean BMD hip (g/cm^**2**^)	Mean BMD femur neck (g/cm^**2**^***)***
Anastasilakis et al. 2015 [56]	*Osteoporos Int*	12	1000	800	Denosumab	IM	32	63	28.80	0.97		
					Zoledronate	IV	26	63	28.70	0.94		
Black et al. 2006 [57]	*J Am Med Ass*	60	655		Alendronate	OS	329	73	25.70	0.90	0.73	0.62
			667		Alendronate	OS	333	73	25.90	0.89	0.73	0.61
			635		Placebo	OS	437	74	25.80	0.90	0.72	0.61
Black et al. 2015 [58]	*J Bone Min Res*	36	1000–1500	400–1200	Zoledronate	IV	95	78	24.60		0.69	0.58
					Placebo	IV	95	78	25.00		0.71	0.58
Brown et al. 2014 [9]	*Osteoporos Int*	12			Denosumab	SC	852	68				
					Ibandronate	OS	851	67				
					Risedronate	OS						
Chesnut et al. 2004 [59]	*J Bone Min Res*	36	500	400	Ibandronate	OS	977	69	26.20			
					Ibandronate	OS	977	69	26.20			
					Placebo	OS	975	69	26.20			
Chung et al. 2009 [10]	*Calcif Tissue Int*	6	500	125	Ibandronate/risedronate	OS	176	61	23.30			
					Risedronate/ibandronate	OS	176	62	23.40			
Cosman et al. 2011 [60]	*J Bone Min Res*	12	1000–1200	400–800	Zoledronate/teriparatide	IV/SC	137	65	25.30	0.74	0.71	
					Zoledronate	IV	137	66	25.30	0.72	0.68	
					Placebo/teriparatide	IV/SC	138	64	25.30	0.73	0.71	
Cosman et al. 2016 [61]	*New England J Med*	12	500–1000	600–800	Romosozumab	SC	3589	71				
					Placebo	SC	3591	71				
		24	500–1000	600–800	Denosumab	SC	3589	71				
					Denosumab	SC	3591	71				
Gonnelli et al. 2014 [62]	*Bone*	12	841	400	Zoledronate	IV	30	66	26.10	0.82	0.79	
			870		Ibandronate	IV	30	67	25.70	0.82	0.79	
Greenspan et al. 2015 [63]	*J Am Med Ass*	24	807	163	Zoledronate	IV	89	85	28.20	0.93	0.68	0.61
			763	168	Placebo	IV	92	86	26.90	0.97	0.70	0.62
Grey et al. 2009 [64]	*J Clin Endocrinol Metab*	24	935		Zoledronate	IV	25	62		1.06	0.85	
			916		Placebo	IV	25	65		1.03	0.86	
Grey et al. 2012 [65]	*J Clin Endocrinol Metab*	12	960		Zoledronate	IV	43	64		1.01	0.85	
			880		Zoledronate	IV	43	66		1.03	0.84	
			850		Zoledronate	IV	43	66		1.05	0.84	
			950		Placebo	IV	43	65		1.03	0.87	
Guanabens et al. 2013 [11]	*Hepatology*	24	1000		Ibandronate	OS	14	65	26.60	0.90	0.84	0.79
					Alendronate	OS	19	63	26.60	0.88	0.81	0.77
Hooper et al. 2005 [66]	*Climacteric*	24			Risedronate	1OS	128	53		1.08		
					Risedronate	OS	129	53		1.08		
					Placebo	OD	126	53		1.08		
Kendler et al. 2019 [67]	*Osteoporosis Int*	12	>1000	>800	Romosozumab	SC	16	69				
					Romosozumab	SC	19	68				
					Romosozumab	SC	14					
					Romosozumab	SC	12					
Iwamoto et al. 2008 [68]	*Yonsei Med J*	12	800		Alendronate	OS	61	70	21.90	0.62		
					ECT	OS	61	69	21.70	0.65		
Iwamoto et al. 2011 [69]	*Osteoporosis Int*	6	800		Alendronate	OS	97	78	22.00			
					Raloxifene	IM	97	82	21.90			
Leder et al. 2015 [13]	*The Lancet*	48			Teriparatide-denosumab	SC	27	66	25.50	0.82		0.64
					Denosumab-teriparatide	SC	27	65	23.80	0.86		0.64
					Combined-denosumab	SC	23	65	25.90	0.85		0.64
Leder et al. 2014 [70]	*J Clin Endocrinol Metab*	24			Teriparatide	SC	31	66	25.50	0.82		0.64
					Denosumab	SC	33	66	24.10	0.87		0.64
					Combined	SC	30	66	25.40	0.86		0.64
Liang et al. 2017 [71]	*Orthop Surg*	24			Zoledronate	IV	155	57	21.80	0.63	0.75	
					Placebo	IV	95	57	21.60	0.63	0.75	
Lufkin et al. 1998 [72]	*J Bone Min Res*	12			Raloxifene	OS	48	67	24.80	0.75	0.64	
					Raloxifene	OS	47	67	26.20	0.81	0.69	
			750	400	Calcium/vit D	OS	48	68	25.30	0.77	0.67	
McClung et al. 2014 [73]	*New England J Med*	12	1000	800	Romosozumab	SC	44	67				
					Romosozumab	SC	46	67				
					Romosozumab	SC	49	67				
					Romosozumab	SC	52	67				
					Romosozumab	SC	53	67				
					Alendronate	OS	47	67				
					Teriparatide	SC	46	67				
					Placebo	SC	47	67				
McClung et al. 2009 [74]	*Obstet Gynecol*	24	500–1200	400–800	Zoledronate	IV	181	60	26.50	0.86		0.69
					Zoledronate-placebo	IV	154	60	27.30	0.86		0.69
					Placebo	IV	188	61	27.20	0.86		0.69
McClung et al. 2018 [75]	*J Bone Min Res*	12	1000	800	Denosumab	SC	127	67				
					Placebo	SC	131	67				
Meunier et al. 2004 [76]	*New England J Med*	36	1000	400–800	Strontium ranelate	OS	719	69	26.20	0.73	0.69	0.59
					Placebo	OS	723	69	26.20	0.72	0.68	0.59
Meunier et al. 2009 [77]	*Osteoporos Int*	12	1000	400–800	Strontium ranelate	OS	221	72		0.85		0.66
					Strontium ranelate	OS	434	72		0.72		0.58
					Placebo	OS	225	72		0.86		0.64
Miller et al. 2016 [14]	*J Clin Endocrinol Metab*	12	1000	800	Denosumab	SC	321	69	24.30			
					Zoledronate	IV	322	70	24.30			
Morii et al. 2003 [78]	*Osteoporos Int*	13			Raloxifene	OS	90	65	21.50	0.66		
					Raloxifene	OS	93	65	21.90	0.67		
					Placebo	OS	97	64	22.00	0.64		
Paggiosi et al. 2014 [79]	*Osteoporos Int*	24	1200	800	Alendronate	OS	57	68	25.90	0.79	0.75	0.64
					Ibandronate	OS	58	67	26.40	0.80	0.78	0.64
					Risedronate	OS	57	67	26.80	0.81	0.80	0.67
					Control		226	38	25.10	1.07	0.97	0.86
Papapoulos et al. 2012 [80]	*J Bone Min Res*	24			Denosumab	SC	2343	75				
					Denosumab	SC	2207	75				
Recknor et al. 2013 [81]	*Obstet Gynecol*	12	500	800	Denosumab	SC	417	67	25.50			
					Ibandronate	OS	416	66	25.10			
Saag et al. 2017 [82]	*New England J Med*	24			Alendronate	OS	2047	74	25.40			
					Romosozumab-alendronate	SC-OS	2046	74	25.50			
Sanad et al. 2011 [83]	*Climacteric*	12	1500	400	Raloxifene	OS	35	63	26.50	0.73	0.69	0.63
					Alendronate	OS	31	62	25.80	0.75	0.72	0.63
					Raloxifene/alendronate	OS	32	63	26.30	0.75	0.71	0.64
Tsai et al. 2013 [84]	*Lancet*	12			Teriparatide	SC	31	66	25.50	0.82	0.76	0.64
					Denosumab	SC	33	66	24.10	0.87	0.77	0.64
					Teriparatide/denosumab	SC	30	66	25.40	0.86	0.76	0.64
Tsai et al. 2019 [85]	*Lancet*	15			Teriparatide-denosumab	SC	35	66	23.00	0.83	0.74	0.65
					Teriparatide-denosumab	SC	34	67	22.80	0.79	0.74	0.62

### Outcomes of interest

Greater values of bALP results associated with a greater rate of vertebral fractures (P = 0.001; *r* = 0.8), non-vertebral fractures (P = 0.0001; *r* = 0.7), overall BMD (P = 0.01; *r* = −0.8), BMD hip (P = 0.04; *r* = −0.5), and BMD femur (P = 0.003; *r* = −0.9) at baseline. No association with bALP at baseline and other endpoints at follow-up was found. Greater values of NTx were associated with lower T score of the spine (P = 0.03; *r* = −0.7) and of the hip (P = 0.04; *r* = −0.7) at baseline. Greater values of NTx at baseline were associated with a greater rate of adverse events at the last follow-up (P = 0.02; *r* = 0.9). Greater values of CTx were associated with lower BMD spine (P = 0.04; *r* = −0.3), BMD hip (P = 0.01; *r* = 0.5), and BMD femur (P = 0.0007; *r* = 0.6) at baseline. Greater values of CTx at baseline were associated with a greater rate of adverse events leading to discontinuation (P = 0.04; *r* = 0.5), gastrointestinal adverse events (P = 0.0001; *r* = 0.7), musculoskeletal adverse events (P = 0.04; *r* = 0.4), and mortality (P = 0.04; *r* = 0.6). Greater values of PINP were associated with lower BMD at baseline (P = 0.008; *r* = −0.4). Greater values of PINP at baseline were associated with a greater rate of gastrointestinal adverse events (P = 0.02; *r* = 0.6) at the last follow-up. No further statistically significant associations were found. Table 2 shows the overall results of the multivariate analyses.

**Table 2:** Overall results of the pairwise correlations

Endpoint	bALP		NTx	CTx		PINP		
	***P***	***r***	***P***	***r***	***P***	***r***	***P***	***r***
*Baseline*								
Vertebral fractures	*0.0001*	*0.8*	0.3	0.3	0.6	0.1	0.4	0.2
Non-vertebral fractures	*0.01*	*0.7*	0.1	0.9	0.8	−0.1	0.3	0.2
BMD	*0.01*	*−0.8*	0.5	0.4	0.1	0.5	*0.008*	*−0.4*
BMI	0.9	0.0	0.09	−0.4	0.4	−0.3	0.2	−0.2
BMD spine	0.2	−0.3	0.6	0.2	*0.04*	*−0.3*	0.5	−0.1
BMD hip	*0.04*	*−0.5*	0.9	−0.1	*0.01*	*0.5*	0.06	0.4
BMD femur	*0.003*	*−0.9*	0.2	−0.5	*0.0007*	*0.6*	0.2	0.4
T score spine	0.4	−0.3	*0.03*	*−0.7*	0.5	−0.1	0.6	0.1
T score femur	0.07	0.5	0.08	−0.8	0.09	0.3	0.5	0.1
T score hip	0.1	1.0	*0.04*	*−0.7*	0.3	0.2	0.8	0.0
*Follow-up*								
BMD spine	0.9	0.0	0.4	0.3	0.4	0.1	0.3	0.2
BMD hip	0.2	0.3	0.9	0.1	0.3	0.2	0.3	0.2
BMD femur	0.3	0.3	0.9	0.0	0.3	0.4	0.3	0.3
Body height	1.00	−1.0	0.1	−1.0	0.1	1.0	0.1	1.0
Non-vertebral fractures	0.3	−0.3	0.1	1.0	0.4	−0.2	0.7	−0.1
Vertebral fractures	0.5	−0.2	0.7	-0.2	0.3	−0.9	0.3	0.2
Hip fractures	1.00	1.0			1.0	−1.0		
Femur fractures	0.1	−1.0			0.07	−0.7	0.1	−1.0
Adverse events	0.9	0.0	*0.02*	*0.9*	0.1	0.2	0.9	0.0
Serious adverse events	0.1	−1.0	0.9	0.2	0.1	0.3	0.5	0.2
Adverse events leading to discontinuation	0.1	0.6	0.3	−0.4	*0.04*	*0.5*	0.4	0.2
Gastrointestinal adverse events	0.3	−0.6	0.3	0.3	*0.0001*	*0.7*	*0.02*	*0.6*
Musculoskeletal adverse events	0.8	−0.1			*0.04*	*0.4*	0.4	0.2
Osteonecrosis					0.9	−0.1	0.4	−0.4
Mortality	1.00	1.0	0.93	0.1	*0.04*	*0.6*	0.1	0.5

## Discussion

According to the systematic review, all BMTs analyzed were useful to monitor the effects of pharmacological therapy setting in postmenopausal osteoporosis. Greater values of bALP have been associated with vertebral fractures and non-vertebral fractures with overall BMD, hip BMD, and femur BMD at baseline. Furthermore, greater values of NTx were associated with lower T score of the spine and of the hip at baseline. Greater values of NTx at baseline were also associated to adverse events at the last follow-up. CTx showed interesting associations, too: greater values were associated to lower spine, hip, and femur BMD at baseline. Greater values of this BMT at baseline were also associated to a greater rate of adverse events leading to discontinuation, gastrointestinal adverse events, musculoskeletal adverse events, and mortality. Finally, greater values of PINP were associated to lower BMD at baseline. High values at baseline have been associated to gastrointestinal adverse events at the last follow-up. Because of their ability to provide information about rapid changes in bone turnover, BMTs have been the subject of numerous studies to investigate their possible role in the management of osteoporotic patients [17, 18, 27]. Bone turnover is a dynamic process which involves bone resorption and bone formation [28, 29]. Several bone turnover markers have been highlighted in clinical practice [27, 30, 31], although not to necessarily identify better therapy outcomes.

Markers of bone formation and resorption have been classified [17]. BALP and PINP are considered bone formation markers [32]. BALP is a membrane-bound enzyme produced by osteoblasts, positively correlated with bone formation [17, 33]. Its role in identifying the risk of fracture has been highlighted [34] when Bjarnason et al. first demonstrated the relationship between the modification of the values of this BMT and the risk of fracture [17, 33]. Statistically significant associations between bALP levels and fracture risk have been also analyzed showing possible association with numerous BMTs [35]. However, the association was not statistically significant, which was not the case for osteocalcin (OC), PINP, CTx, and NTx [35]. In a Japanese population, in contrast, bALP did predict vertebral fractures [36]. The association between bALP levels and BMD was instead analyzed in adults with and without diabetes [37]. In non-diabetic subjects, bALP levels were associated to BMD [37]. On the other hand, there was no relationship between bALP and BMD in elderly men with no history of fractures [38]. Procollagen type 1 N-terminal propeptide (PINP) derives from the type 1 collagen formation process, from its precursor, procollagen [17, 39]. It is considered a standard indicator of bone formation [27]. Kučukalić-Selimović et al. analyzed the role of this BMT in the bone status assessment and found a significant negative correlation between BMD (at the femoral neck, total hip, and lumbar spine) and serum levels of PINP [40].

NTx and CTx are considered markers of bone resorption [17]. These two BMTs are two different forms of a telopeptide of type I collagen, acting in the collagen degradation process, and are found in serum and in urines [41–43]. NTx showed an association with the T-score spine and hip levels at baseline, while greater CTx values were associated with lower spine, hip, and femur BMD at baseline. Since they are markers of resorption, their levels may increase in increased bone turnover, leading to a reduction in BMD and T-score. Indeed, high bone turnover setting (hyperthyroidism, hyperparathyroidism, and Paget disease) is associated with greater values of BMTs [44–49]. This has also been reported in postmenopausal women when a reduction of BMD may be appreciable [50, 51]. Although CTx and PINP have been recommended as the reference standard for bone resorption and bone formation [27], in the light of the results of this systematic review, all BMTs can be statistically related to specific complications.

This study showed several limitations, as data were based on a large population, hence they carry a high risk of bias. There is still little literature available about the actual therapeutic role for these BMTs. In fact, the studies analyzed in this review did not evaluate BMTs as primary outcomes. The pathophysiology of these markers and their relationship with osteoporosis complications should be analyzed more specifically, as they could have marked clinical potential. Future studies should evaluate whether osteoporosis complication can be predicted from variation of a given BMT, and, subsequently establish which drug could be suitable for a specific individual. These substances can be measured in serum or urine by immunological tests [52, 53], and their levels are influenced by endogenous and exogenous factors [17, 19, 31, 54, 55]. As differences in sampling methods still remain, specific research groups highlighted the need for standardization of the collection method [27]. Another important limitation of this review is the heterogeneity of the studies evaluated, as they analyzed the intervention of different types of drugs, or the same drugs with different dosages. Furthermore, daily vitamin D administration was not homogeneous in all studies. Finally, future studies should consider to standardize the measurement methods of BMTs.

## Conclusion

The present systematic review shows that further studies should validate the use of BMTs in clinical practice. Our analysis supports the adoption of BMTs during pharmacological therapy setting of patients with postmenopausal osteoporosis. Further studies are required to analyze their role in predicting complications as a primary outcome.

## Data Availability

This study does not contain any third material.

## Abbreviations

SD: Standard deviation
IQR: While median and interquartile range
RCTs: Randomized clinical trials
OCEBM: Oxford Centre of Evidence-Based Medicine
BMD: Bone mineral density
BMI: Body mass index
BTMs: Biochemical markers of bone turnover
bALP: Bone alkaline phosphatase
PINP: Procollagen type I N propeptide
bCTx: Serum cross-linked C-telopeptides of type I collagen
NTx: Urinary cross-linked N-telopeptides of type I collagen
